# Organic and Inorganic Dyes in Polyelectrolyte Multilayer Films

**DOI:** 10.3390/ma5122681

**Published:** 2012-12-10

**Authors:** Vincent Ball

**Affiliations:** 1 Institut National de la Santé et de la Recherche Médicale, UMR 1121, 11 rue Humann, Strasbourg Cédex 67085, France; E-Mail: vball@unistra.fr; Tel.: +33-3-90-24-32-58; Fax: +33-3-90-24-33-79; 2 Faculté de Chirurgie Dentaire, Université de Strasbourg, 1 Place de l'Hôpital, Strasbourg 67000, France; 3 Fédération de Médecine Translationelle de Strasbourg, 1 Place de l'Hôpital, Strasbourg 67000, France

**Keywords:** dyes, quantum dots, polyelectrolyte multilayer films, step-by-step deposition

## Abstract

Polyelectrolyte multilayer films are a versatile functionalization method of surfaces and rely on the alternated adsorption of oppositely charged species. Among such species, charged dyes can also be alternated with oppositely charged polymers, which is challenging from a fundamental point of view, because polyelectrolytes require a minimal number of charges, whereas even monovalent dyes can be incorporated during the alternated adsorption process. We will not only focus on organic dyes but also on their inorganic counterparts and on metal complexes. Such films offer plenty of possible applications in dye sensitized solar cells. In addition, dyes are massively used in the textile industry and in histology to stain textile fibers or tissues. However, the excess of non bound dyes poses serious environmental problems. It is hence of the highest interest to design materials able to adsorb such dyes in an almost irreversible manner. Polyelectrolyte multilayer films, owing to their ion exchange behavior can be useful for such a task allowing for impressive overconcentration of dyes with respect to the dye in solution. The actual state of knowledge of the interactions between charged dyes and adsorbed polyelectrolytes is the focus of this review article.

## 1. Introduction

**Scheme 1:**
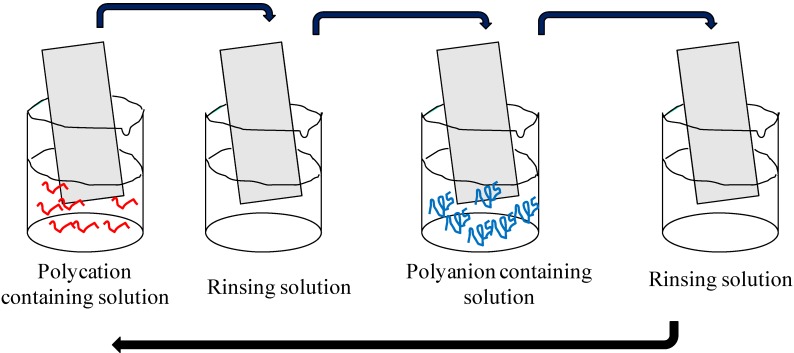
Representation of the deposition steps allowing for the obtention of polyectrolyte multilayer films. The polycation or the polyanion containing solution can be replaced by a solution containing a charged dye or charged nanoparticles. The four steps represented correspond to “one deposition cycle” and can be repeated as many times as required. In the case of alternated spray deposition or alternated spin coating, the dipping steps are replaced by spraying or spin coating steps respectively.

Dyes have been actively used for painting and coloring of textiles for millenaries. These activities consist of coating surfaces to modify their surface properties along with their esthetic appearance. With modern technology it is mandatory to confer some well designed optical properties, like controlled absorbance at specific wavelengths for artificial photosynthesis, non linear optical properties, the possibility to inject charges in semi-conductors for dye sensitized solar cells to state of the art thin films of well controlled thickness. Among such technologies, the coating of surfaces with films prepared according to the “Layer-by-layer” process emerged about 20 years ago [1], after some initial trials in the 1960s [2], as a versatile method to deposit films of well controlled thickness on charged or uncharged substrates. The deposition of such coatings relies on electrostatic, hydrogen bonding [3], charge transfer interactions [4], specific interactions between species carrying mutually complementary binding sites and host-guest interactions [5]. Such films are, most of the time, obtained by alternatively dipping the substrate in solutions containing the species to be deposited. Two successive immersion steps are separated by a rinsing step (Scheme 1) allowing to for the removal of the weakly bound species from the substrates to be coated. The deposition of the two interacting species constitutes a full deposition cycle which can (most of the time, but not systematically) be reiterated for any desired number of times leading to a fine control in the film thickness. Alternated spray coating [6] and alternated spin coating [7] become more and more popular and allow for speeding up of the deposition process with respect to the alternated dipping method. There are more and more “unconventional” ways to produce such kinds of films [8].

The most investigated case corresponds to oppositely charged polyelectrolytes, and in this case, the obtained coatings are called “polyelectrolyte multilayer films” (PEMs). Even when relying on electrostatic interactions, which can be of very peculiar origin due to counter-ion release [9,10], the incorporated species in the “layering” process can be charged nanoparticles, clays, nanotubes. The general principles and physicochemical basis of films deposited in a step-by-step manner have been extensively reviewed [11,12,13,14]. As a peculiar and often neglected example, dyes can also be incorporated in such films. No overall view of such dye containing films is available to date, even if an excellent review describes, among other aspects, layered films containing porphyrins [15]. Hence, it is the aim of the first part of this review to give an overview of the deposition mechanism of such films and to describe the interactions between the incorporated dyes leading to their aggregation process. Such films have found applications in electroluminescent devices and for non linear optical devices. In addition, we will also describe films prepared according to the layer-by-layer deposition method and incorporating inorganic dyes and photoactive supramolecules.

The term layer-by-layer coatings is often misleading because there are more and more cases where the distribution of the complementary entities used to build-up the films do not reflect their position at the moment of the deposition process. This comes from intermixing processes, diffusion of species through the whole thickness of the films is even one possible mechanism to explain supralinear increase in the films thickness with the number of deposition cycles. Such films allow for the incorporation of dyes and nanoparticles in a post deposition manner and could offer new opportunities for drug release [16] as well as for depolution of water.

## 2. Use of Charged Organic Dyes in the Layering Process

### 2.1. Films Made In a Step-By-Step Manner and Incorporating Organic Dyes

Very early in the history of polyelectrolyte multilayer films, a dye conjugated to a polycation was deposited at different stages of the deposition but always in the same polyelectrolyte environment in order to probe the local pH inside the film, hence at different distances from the film solution interface and depending on the last deposited polyelectrolyte (either the cationic poly(allylamine hydrochloride) (PAH) or the anionic poly(sodium-4-styrene sulfonate) (PSS). Indeed the fluorescenece emission spectrum of the used dye, fluorescein depends markedly on the local pH value in the film [17]. In this investigation, fluorescence emission was measured in a total internal reflection configuration and allowed to calculate a potential distribution from the film-solution interface in the “bulk” of the film. In turn, the exponential decrease of the electrostatic potential from the film-solution interface in the film allowed for the calculation of the value of an apparent Debye screening length in the material of the film as compared to the solution with which the film was in contact. This important paper, using PAH labeled with FITC (fluorescein isothiocyanate) as a probe, is one of the most important articles, with those showing the partial interpenetration of adjacent layers [18], which established the physicochemical basis of polyelectrolyte multilayer films. Notably, the article by Klitzing and Möhwald [17] distinguished the film-solution interface from the “bulk” of the film. In turn, the build-up of the films is also influenced by the substrate. Hence, a first order “picture” of polyelectrolyte multilayer films consist to distinguish three zones: the substrate-film interface, the “bulk” of the film and the film-solution interface [19]. The internal “bulk” region can only appear when the films are thicker than a critical value.

The first examples of SBS deposition using an anionic dye as the negatively charged counterpart and an oppositely charged polycation, poly(L-lysine), has been described by Crane *et al.* [20]. This work was followed shortly by the investigation published by Wrighton *et al.* [21]. The first article showed the possibility to obtain films of increasing thickness with the number of deposition steps using poly(L-lysine hydrobromide) (PLL) as the polycation and either congo red (CR) or copper (II) phtalocyanine tetrasulfonic acid (CPTA) as the anionic dyes. The dyes forced PLL to adopt mainly a α helical conformation in the films whereas the polycation was in the form of a random coil in free solution. CR displayed a positive dichroism in its π–π* transition at around 500 nm whereas CPTA displayed a negative circular dichroism in its Q band (550–750 nm). Linear dichroism experiments also showed that the dipolar axis of CR lies preferentially along the dipping axis of the quartz slide suggesting that the dyes align along the direction of the main shear forces applied during the film deposition. The investigation by Wrighton *et al.* is also particularly interesting because it shows the possibility of building up films by alternating the adsorption of two dyes: A cationic tetraruthenated zinc porphyrin and the anionic meso tetraphenylporphyrin sulfonate [21]. The obtained films were electrochemically active up to the deposition of 30 deposition cycles, owing to the presence of Zn in the tetraruthenated zinc porphyrin. These films were also able to catalyze the reduction of O_2_ in water [21]. Similarly, Rubner *et al.* deposited LBL films incorporating two anionic dyes, Ponceau SS and Infra red dye 125 in a quadrolayer deposition sequence to obtain (PAH-Ponceau SS-PAH-Infra red dye 125)_5_ films exhibiting the characteristic absorption peaks of both dyes [22].

**Scheme 2:**
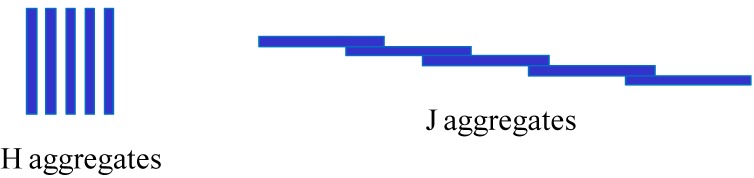
Schematic structure of H and J aggregates formed by dyes in the condensed phase. Each individual dye molecule (or ion) is represented by a blue rectangle.

The preferential orientation of the J aggregates (see Scheme 2 for the definition of J and H aggregates) was also investigated in films made from the alternate adsorption of poly(diallyldimethyl ammonium chloride) (PDDA) and tetrakis(4-sulfonatophenyl) porphyrin diacid by means of linear dichroism. The transition dipole of the J aggregates was found to lie parallel to the film surface [23].

**Figure 1:**
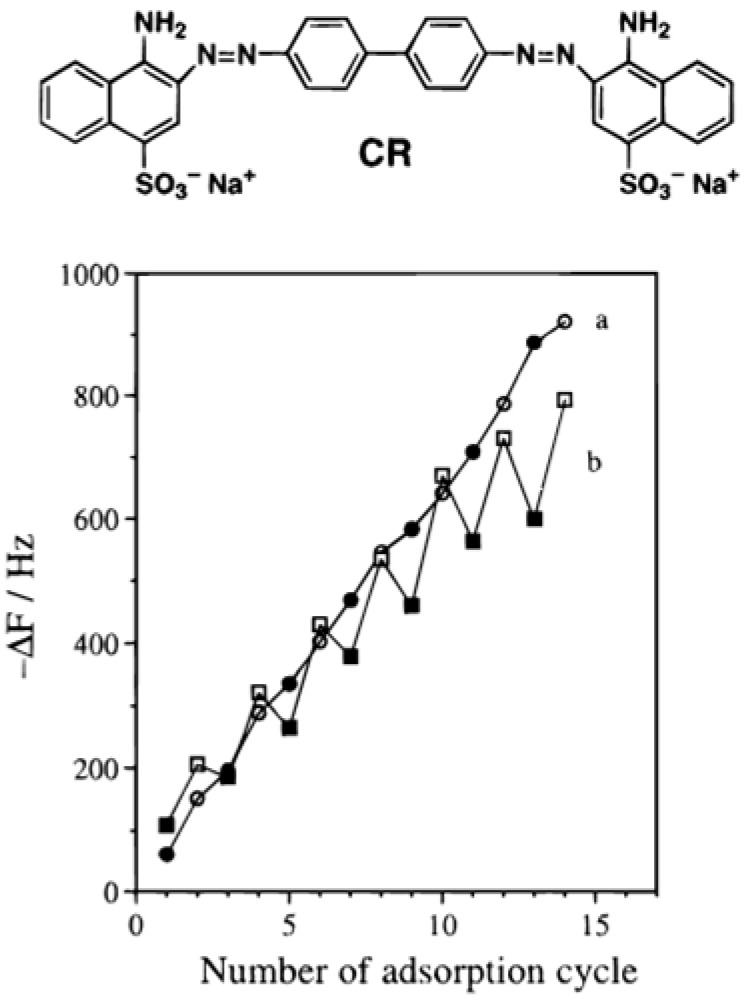
Monitoring of the frequency decrease during the deposition of (CR-PDDA)_*n*_ films (curve a, ○,●) and during the deposition of (CR-PEI)_*n*_ films (curve b, □,■). The empty symbols correspond to the deposition of the dye congo red (CR, whose structure is shown) and the filled symbols correspond to the deposition of the polycation. The dye containing films were deposited on a (PSS-PEI)_4_ cushion acting as a precursor film. Reproduced with permission from [24]. Copyright 1997 the American Chemical Society.(

However, the first complete characterization of SBS films made from charged dyes and polycations has been published by the group of Kunitake [24]. Two major findings are described in this investigation: the occurrence of partial dye desorption upon subsequent adsorption of the polyelectrolyte and the aggregation of the dyes (mainly in the form of J aggregates). The zigzag like adsorption curves, with adsorption-desorption phenomena, observed by following the alternated adsorption of the dye (congo red, CR) and the polycation (poly(ethyleneimine)) (PEI) by means of quartz crystal microbalance (Figure 1) can be reduced by decreasing the solution concentration of both the anionic dye and the polycation. This observation as well as the formation of J aggregates upon the adsorption of dyes is typical of films obtained by LBL deposition with dyes and has been reported many times after the work of Kunitake *et al.*

**Figure 2:**
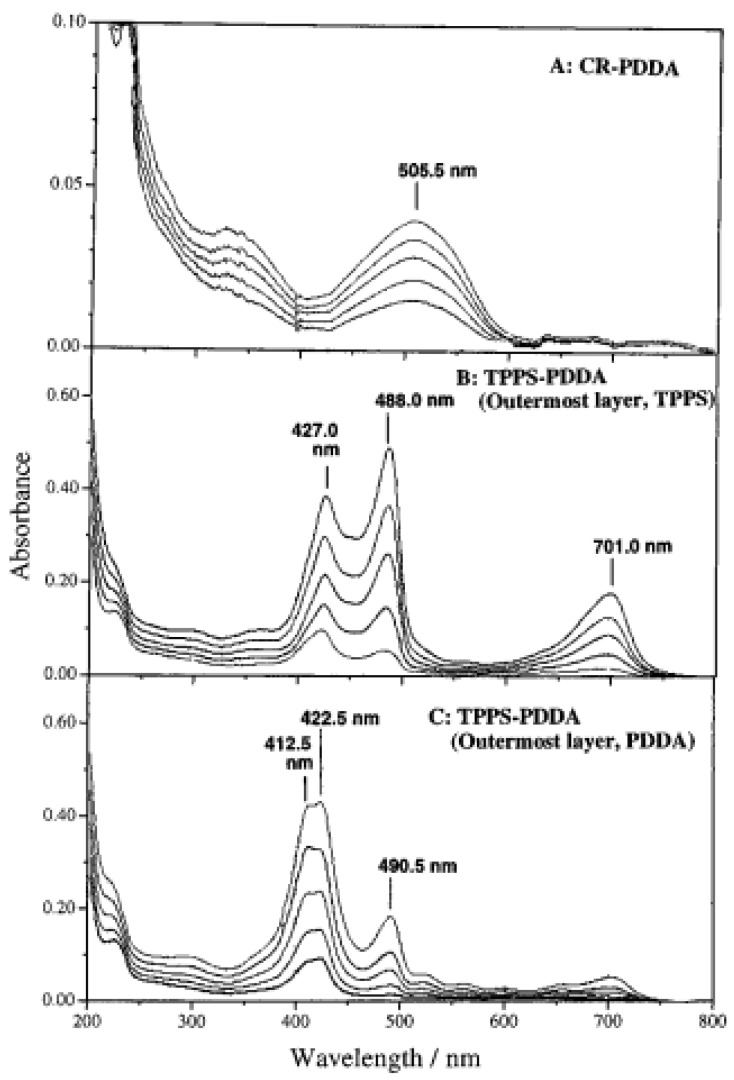
UV visible spectra of SBS films made wih CR and PDDA (A), with TPPS and PDDA (B and C). In B, the deposition process was ended with TPPS whereas it ended with PDDA in C. Reproduced with permission from [24]. Copyright 1997 the American Chemical Society.

Complementary, the UV-visible spectrum of the films made from tetraphenylporphyrinetetrasulfonic acid (TPPS) and PDDA also showed an interesting even-odd effect with marked spectral changes depending on the nature of the last deposited compound (Figure 2). The same phenomenon of dye release upon subsequent adsorption of the polycation, poly(allyl amine hydrochloride) (PAH) was found for pyrenetetrasulfonic acid (4-PSA) [25]. The amount of adsorbed as well as the amount of desorbed dye was markedly dependent on the ionic strength of the solution into which the PAH solution was prepared, but the amount of irreversibly bound 4-PSA was almost ionic strength independent [25].

When the diazo dye Direct Red 80, dissolved in pure water, was deposited in alternation with a polycation having 95% of *N*,*N*-dimethyl-2-hydroxypropyleneammonium chloride units and in the presence of either NaCl or NaBr (0.2 or 1.0 M), the aggregation state of the dye is not affected by the ionic strength. However, the amount of deposited dye is higher in the presence of NaBr than with NaCl at a given ionic strength. This is in direct relationship with the effect of the salt on the reduced viscosity of the polyelectrolyte solution and hence of the hydrodynamic diameter of the polyelectrolyte [26].

Films made from the di-sodium salt of fluorescein and strong polycations like poly(diallyldimethyl ammonium chloride) (PDDA) or poly(vinyl benzyl chloride) quaternized with *N*,*N*-dimethyl ethanolamine (PVBAC) have been successfully deposited despite the low charge of dye, 2 elementary charges [27]. The same holds true for the deposition of the anionic squarylium dye and the cationic polyelectrolyte PDADMAC [28]. Usually the layer-by-layer deposition is not possible when using polyelectrolytes of weak charge density [29]. In the case of charged dyes, even if the total number of elementary charges is low, the charge density is high and it is well possible that hydrophobic interactions between the hydrophobic moieties of the organic dyes and those carried by the polyelectrolytes significantly contribute to the driving force allowing for spontaneous self assembly. This was shown by the fact that the absorbance of the films as well as their thickness was higher in the case where the polyelectrolyte carried an aromatic ring (PVBAC). Interestingly, the maximal absorbance of the (PVBAC-dye)_10_-PVBAC films decreased with storage time in air and the position of the absorption maximum underwent a blue shift from 518 to about 505 nm indicating a possible lower aggregation of fluorescein upon storage time [27].

The aggregation state of 5,10,15,20-Tetrakis(4-sulfonatophenyl) porphyrins (SP) in (PAH-SP)*_n_* films depends markedly on the change in solution pH after film deposition [30]. UV-vis spectroscopy showed that the porphyrins undergo a transition from the J state (edge to edge aggregation, Scheme 2) at low pH to an H aggregation state (face to face aggregates) at pH above 3.

Monovalent and hydrophobic azulenes were alternated with sodium polystyrene sulfonate to produce smooth and photoluminescent coatings [31]. Surprisingly, the position of the absorption maxima of the dyes as well as the position of the emission maxima were identical in the film state and in solution.

In the same order of ideas, but of major practical interest for light emitting devices, oligomers of Ru(bpy)_3_^2+^ were deposited in an LBL manner with various polyanions including sulfonated poly(p-phenylene), PSS, poly(methacrylic acid) (PMAA) and poly(acrylic acid) (PAA) [32]. The current-voltage and the light-voltage characteristics of the films made from the weak polyelectrolytes were reversible whereas the films made from the sulfonated polyanions displayed a marked diode behavior.

Films made from the alternated deposition of azobenzene dyes, for instance DR80, and polycations can undergo interesting photoalignment under irradiation with linearly polarized UV light [33,34].

Films obtained on cotton fabrics through the alternated adsorption of PDDA and three anionic fluorescence brightening agents were shown to improve the UV protection factor (in the UV-B range, *i.e.*, from 280 to 320 nm and in the UV-A range, *i.e.*, from 320 to 400 nm) of these fibers. In addition the coatings were shown to be adherent on cotton for at least five home washings which is mandatory for practical applications of a modified textile [35].

As a peculiar dye, eumelanin grains, the natural pigment of the skin which are negatively charged at pH = 12 at which the experiment was performed, could be deposited in alternation with PDDA to yield a film with a Donnan potential of −70 mV and having a measurable permeability for hexacyanoferrate anions [36]. In comparison, a pure melanin film deposited from a dopamine solution using Messersmith’s method [37] (oxygenation of a dopamine solution in the presence of Tris buffer at pH 8.5) was totally impermeable for this redox probe as soon as the coating thickness was higher than 10 nm.

### 2.2. Films with Ordered Dye Containing Layers

Most of the investigations concerning the incorporation of dyes in films deposited in a layer-by-layer manner were of fundamental nature, with the aim to determine the concentration of the dye in the films, its aggregation state, *etc.* A step towards real world applications in the field of directional energy transfer has been reached by Jonas *et al.* [38]. They deposited a stratified multilayer where the first stack was made of poly(vinylbenzylammonium chloride) (PVBAC) and PSS doped with a dye and the second stack was made from a thin (PAH-PAA)_5_ film. The dye, either pyranine, fluorescein or Nile Blue was adsorbed in the presence of the polycation. The (PAH-PAA)_5_ film was thermally crosslinked to yield amide groups (between the carboxylate groups of PAH and the amine functions of PAH) [39]. These crosslinked films acted as barriers impeding the diffusion of the dye from one (PVBAC-PSS)_10_ compartment to the neighboring one. The close contact of the dyes in a well ordered manner allowed for fluorescence resonance energy transfer from pyranine to fluorescein and from fluorescein to Nile Blue, mimicking a light diode [38].

## 3. Films Made from Polyelectrolytes Covalently Modified with Dyes

If high local and controlled concentration of the dye is required and if one desires to avoid any dye desorption from the film in the solution, as was shown to occur (Figure 1), films can be constructed according to the LBL method by using dyes covalently bound to one of the constituting polyelectrolytes. For instance, polystyrene sulfonate containing ethylene groups modified with pyrene (Py) have been used to construct PEI-(PSS-Py/PDDA)*_n_* films. Such films allow for a vectorial electron transport to polyviologen put in contact with the films, but without noticeable polyviologen diffusion in the polyelectrolyte multilayer film [40]. The absorbance of (PSS-PAH grafted with fluorescein)_10_ films deposited on ITO electrodes was sensitive to the applied voltage above a threshold value of +1.2 V *vs.* Ag/AgCl [41]. This reversible change was explained by a pH change induced by electrolysis of water. Since the oxidation of water is occurring at the electrode film interface, the thinner the films are the more pronounced is the absorption change.

**Figure 3:**
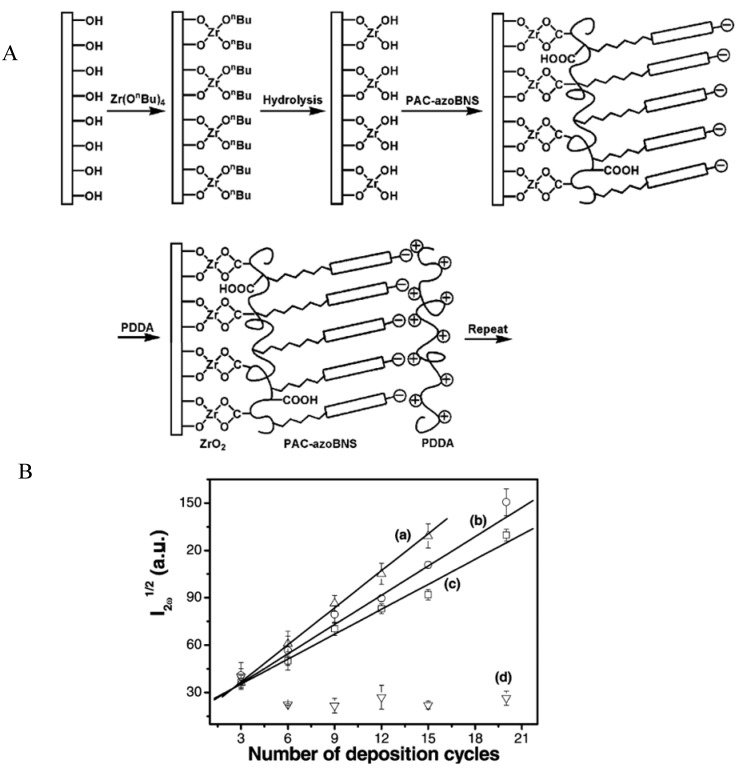
(**A**) Schematic representation of the deposition of a (ZrO_2_/PAC-azoBNS/PDDA)*_n_* multilayer film. (**B**) Evolution of the square root of the second harmonic generation signal as a function of the number of deposition cycles, *n*, for (ZrO_2_/PAC-azoBNS/PDDA)*_n_* films with 62% (a); 44% (b) and 29% (c) of grafting density on the PAC polymer. Curve (d) represents the optical activity of (PDDA/PAC-azoNBS)*_n_* films where the PAC polymer carried 44% of its monomers grafted with the azobenzene chromophore. Reproduced with permission from [42]. Copyright 2004 the American Chemical Society.

Finally, another advantage of using polyelectrolytes grafted with azobenzene sulfonate groups (polyacrylic acid carrying 29, 44 or 62% of azobenzene sulfonate groups, PAC-azoBNS) with respect to azobenzene sulfonate monomers was demonstrated for the generation of layered films displaying non linear optical properties [42]. Interestingly (PDDA/PAC-azoBNS)*_n_* films did not display an increase in the square root of the second harmonic generation signal because PAC-azoBNS deposits both with its carboxylic acid and sulfonate groups on the PDDA layer and hence induces an equiprobable orientation of the azobenzene groups at the film/air and at the film/substrate interface. This random orientation of the chromophore is in opposition with the requirement to have non-centrosymmetrically oriented groups on a sufficiently large domain. To fulfill this requirement, the PAC-azoBNS was deposited on a ZrO_2_ film produced by sol-gel chemistry on the silica substrate or on the PDDA layer. The carboxylate groups of PAC-azoBNS then coordinate to ZrO_2_ inducing a preferred orientation of the sulfonate groups to the film/air interface (Figure 3) [42].

Without taking such precautions to orient the azobenzene dyes, increasing non linear optical (NLO) signal could be obtained on (SPECH-PSS)*_n_* multilayers up to *n* = 36. SPECH corresponds to stilbazolium substituted polyepichlorohydrin. However, after 36 deposition cycles the NLO signal saturated. These films nevertheless exhibited excellent thermal stability since the NLO signal remained unchanged up to 140 °C [43].

In the field of catalytically active films, poly(4-vinyl pyridine) carrying grafted Mn(III) porphyrins were alternatively deposited with PSS to obtain electroactive films acting as catalysts for the decoloration of azo dyes in the presence of hydrogen peroxide [44].

From a pure physicochemical point of view, the incorporation of polyelectrolytes carrying covalently bound fluorophores allows fundamental investigation of the properties of polyelectrolyte multilayer films as already mentioned [17]. In addition, fluorescence recovery after photobleaching allowed to investigate the diffusion coefficients of the PLL polyelectrolytes modified with fluorescein isothiocyanate [45,46]. Using similar molecules but carrying dyes playing the role of energy donors and acceptors, Laschewsky *et al.* investigated Förster energy resonance transfer in self assembled multilayer films [47].

## 4. Films Made with Supramolecular Complexes or Inorganic Dyes

### 4.1. LBL Films Made with Supramolecular Complexes

Films obtained in an LBL manner from an osmium complex and from bis(benzonitrile)dichloropalladium grow in an exponential manner as a function of the number of dipping cycles. The origin of this peculiar growth mechanism, close to that observed for PEM films, is due to the porosity of the network and from the ability of the Pd(II) salt to diffuse in and out the film during its interaction with the osmium complex [48].

### 4.2. Films Prepared in a Layer-by-Layer Manner and Incorporating Inorganic Dyes

Examples of films incorporating quantum dots and polyelectrolytes deposited in a stepwise manner appeared early after the proof of principle of layer-by-layer deposition of oppositely charged polyelectrolytes [49]. These investigations have been reviewed recently [50] and we will not provide too much redundant information. Of particular interest however are the films incorporating TiO_2_ and ruthenium dyes, which showed promising performance as dyes sensitized solar cells [51].

Note that poorly crystalline TiO_2_ nanoparticles can be produced during the LBL deposition process using a polyamine as the polycation and an hydrosoluble complex of Ti^4+^, namely Ti(IV) (bisammoniumlactatato) dihydroxyde, which undergoes hydrolysis and polycondensation when put in contact with the polyamine [52,53]. Proteins can also be employed for such a process [54,55] which is of biomimetic inspiration: indeed diatoms and other marine organisms use cationic proteins, silaffins and silicateins, to induce biomineralization of silica using silicic acid as a precursor [56].

## 5. Diffusion of Dyes in the Films and Release out of the Films

### 5.1. Exponentially Growing Polyelectrolyte Multilayer Films as Reservoirs for Quantum Dots and Dyes

A major finding in the field of PEM films prepared according to the LBL method, was the observation of supralinear growth regimes [57,58], meaning that the derivative of the film thickness *vs.* the number of deposited layer pairs is not constant during the deposition process, as is the usually the case for the so called “linear” growth regime. Hence, in most of the explored films the thickness and the amount of deposited molecules increases in proportion with the number of deposition cycles. The supralinear growth corresponds most often to an exponential increase in the film thickness, up to a critical thickness where the thickness increase turns linear again with the number of deposition steps [59]. The most plausible explanation for such an exponential growth regime is the diffusion of a least one of the used species across the whole thickness of the film [60]. This is possible if the interactions between the interacting species are not too strong. Otherwise, the mobility of the species will be strongly reduced leading to a stratified film where each polyelectrolyte stays at a location close to which it was initially deposited. The major advantage of exponentially growing films with respect to their linearly growing counterparts is that a pretty important thickness, in the µm range, can be quickly reached in a pretty small number of deposition cycles, whereas the typical thickness per deposition cycle is of the order of a few nanometers (depending on physical parameters like the ionic strength, temperature and the pH of the solution in the case of weak polyelectrolytes) in a linear growth process.

**Figure 4:**
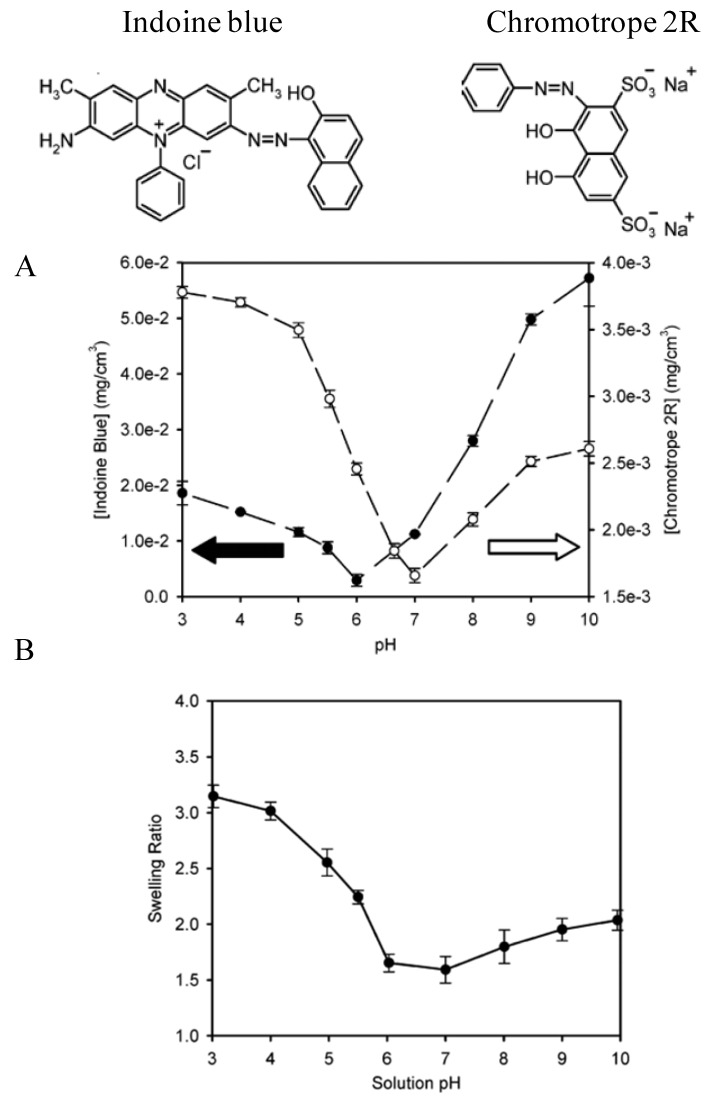
Structure of indoine blue and chromotrope 2R which were allowed to interact during 24 h with (PAH-HA)_10_ films as a function of the solution pH at a constant dye concentration in solution, 0.15 mg mL^−1^. The amount of incorporated dyes is plotted as a function of the solution pH in part B and is seen to correlate with the pH induced film swelling, measured as the ratio of the film thickness at the given pH and the dry film thickness (Part B). Reproduced with permission from [71]. Copyright 2004 the American Chemical Society.

Another difference between linearly and exponentially growing films lies in the difference between their physical properties [61]: Exponentially growing films are highly hydrated [62,63] can undergo important swelling-deswelling processes upon change in external parameters (ionic strength, pH) [64] due to an extrinsic charge compensation of the charge in the “bulk” of such films. This means that electroneutrality is ensured by the preferential incorporation of one type of ions from the electrolyte solution in contact with the film [65]. This property allows such “exponentially growing films” to behave as ion exchange membranes, allowing for instance the incorporation of “colored” redox probes [66,67,68], drugs [69] and dyes [70,71,72,73]. In the investigation published by Burke and Barrett [71], the (PAH-HA)_10_ films, where HA represents hyaluronic acid, were deposited from solutions at pH 3 and the film swelling was investigated under the influence of a pH change. Two dyes, the cationic indoine blue and the anionic chromotrope 2R were also put in contact with the polyelectrolyte multilayer at a constant solution concentration of the dye but under different pH conditions (Figure 4).

It was found that the dye incorporation, which charge was pH independent in the investigated range, correlated with the excess charge of one of the polyelectrolytes in the film. For instance at pH values below 5, HA is partially neutralized through protonation of its carboxylic groups and PAH is strongly positively charged due to protonation of its amino groups, this allows for a strong incorporation of the anionic chromotrope 2R (Figure 4A). The cationic dye indoine blue is strongly incorporated at pH values higher than 5 when HA is strongly charged and PAH partially neutralized. As an interesting finding, the dye incorporation is not only related to the Donnan potential of the film but also to its swelling ratio (Figure 4B) which can be understood on the basis that enough porosity is required for the dye to find available volume in the polyelectrolyte multilayer film. The influence of the film porosity on the incorporation of methylene bluewas is also shown in the investigation of Chung and Rubner [70].

Similarly to the findings of Barrett [71], Sukhishvili *et al.* [74] showed that the positively charged rhodamine 6G binds preferentially to a PMAA_5_ hydrogel film at pH values only above 5 whereas the negatively charged bromphenol blue binds to the film only at pH values below 5. This originates from the fact that the hydrogel was obtained from a hydrogen bonded self assembled multilayer film, made from poly(methacrylic acid) (PMAA) and poly(*N*-vinylpyrrolidone) (PVPON). The hydrogen bonded film was subsequently crosslinked with a carbodiimide activated ethylenediamine crosslinker and finally PVPON was released quantitatively by a pH increase. Hence, the film carried both free amino and free carboxylic acid groups with an excess positive charge at low pH (due to the protonation of both the carboxylic acids and the amino groups) and an excess negative charge at high pH values allowing hence for the preferential binding of negatively and positively charged groups, respectively [74].

**Figure 5:**
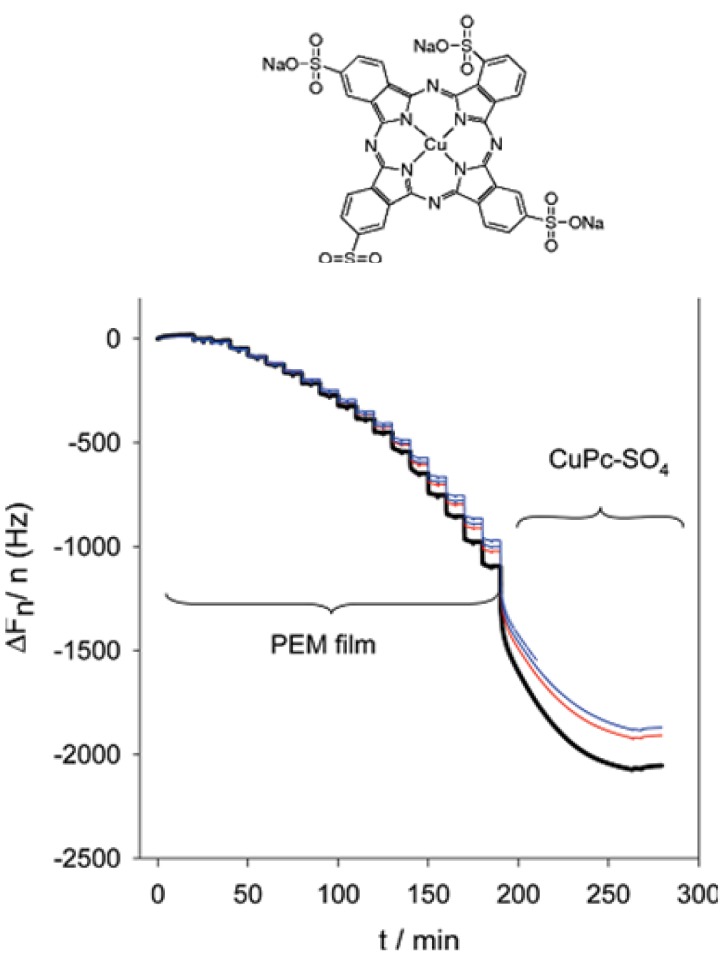
QCM-D experiment allowing to follow *in situ* the deposition of a PEI-(PGA-PLL)_8_ multilayer film followed by its interaction with a 10^−3^ M solution of CuPc-SO_4_ (whose structure is indicated). The experiment was performed in the presence of a 10 mM Tris buffer at pH 7.5 and with additional 150 mM NaCl. Reproduced with permission from [73]. Copyright 2011 the American Chemical Society.

The binding kinetics of cationic and anionic phtalocyanines to (PLL-PGA)*_n_* films, where PGA denotes poly(L-glutamic acid) was investigated by means of quartz crystal microbalance with dissipation (QCM-D) monitoring and was found to be pretty slow (Figure 5). Indeed it took almost 2 h to reach a steady frequency change of the quartz crystal coated with a PEI-(PGA-PLL)_8_ film, of about 100 nm in thickness, put in contact with a 10^−3^ M Cu(II) tetrasulfonato phtalocyanine (Cu-Pc-SO_4_) containing solution [73].

**Figure 6:**
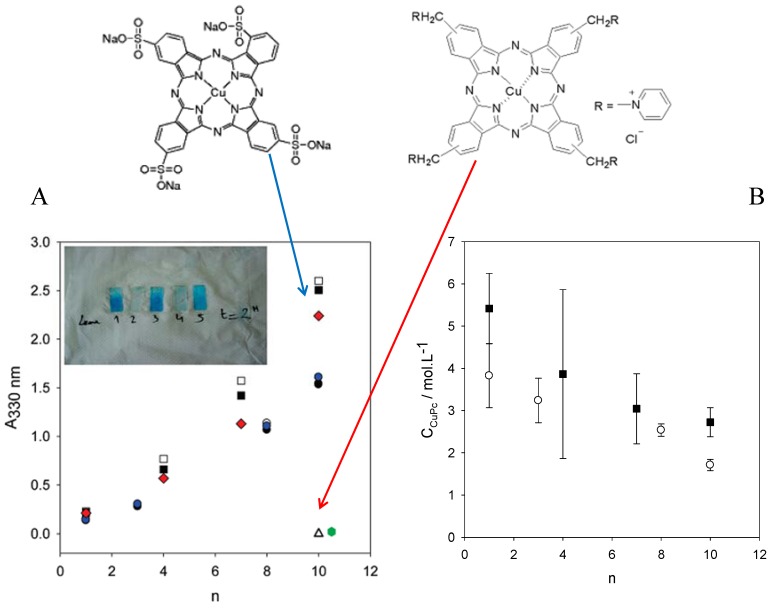
(**A**) Absorption spectra of PEI-(PGA-PLL)_*n*_ films put in contact during 3 h (time sufficient to reach steady state film loading with the dyes) with either the anionic CuPc-SO_4_ or the cationic CuPc-Py (whose structure is indicated). Interaction of CuPc-SO_4_ with PEI-(PGA-PLL)_*n*_ films build-up from MilliQ water (●,○,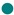, three series of independent experiments), interaction of CuPc-SO_4_ with PEI-(PGA-PLL)_*n*_ films build-up from Tris-NaCl buffer (□,■, two series of experiments) and of the anionic phtalocyanine with PEI-(PGA-PLL)*_n_* –PGA films (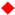). Interaction of CuPc-Py with a PEI-(PGA-PLL)_10_ (△) and with a PEI-(PGA-PLL)_10_-PGA (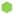) film after 3 h of contact. The inset shows pictures of PEI-(PGA-PLL)_10_ (rows 1 and 2) and of PEI-(PGA-PLL)_10_-PGA (rows 3 and 4) films put in contact with a Tris-NaCl buffer containing CuPc-SO_4_ (1 mM during 2 h, rows 1 and 3) or CuPC-Py (1 mM during 2h, rows 2 and 4). Row 5 corresponds to a PEI-(PGA-PLL)_10_ film put in contact with CuPc-SO_4_ at 10^−4^ M in Tris-NaCl buffer. All the films displayed in the inset were deposited with polyelectrolytes dissolved in water. (**B**) Average concentration of CuPC-SO_4_ and CuPc-Py in PEI-(PGA-PLL)_*n*_ films as a function of the number of deposited ‘‘layer pairs’’: CuPC-SO_4_ in contact with films deposited from polyelectrolyte solutions in Milli Q water (○) and in Tris-NaCl buffer (■). The data for CuPc-Py are not represented here because they correspond to a concentration about 60–70 lower than for CuPc-SO_4_ in the case of films made from *n* = 10 layer pairs. The error bars were calculated from the standard error of the absorbance at 330 nm (3 independent experiments for each value of *n*) and from the standard deviation of the film thickness. Reproduced with permission from [73]. Copyright 2011 the American Chemical Society.

As for the previously considered examples [71,72] the PEI-(PGA-PLL)*_n_* films displayed a strong preferential incorporation (Figure 6) of the negatively charged CuPc-SO_4_ with respect to the cationic CuPc-Py [73]. This finding is in perfect agreement with the expected positive Donnan potential of such films at pH 7.5.

It has to be noted that the average concentration of CuPc-SO_4_ in the investigated polyelectrolyte films is of the order of 2 to 5 mol L^−1^ (Figure 6B), an impressive value when compared to the solution concentration of the dye put in contact with the polyelectrolyte multilayer film, 10^−3^ mol^−1^. Such a high concentration of incorporated species is not uncommon for polyelectrolyte multilayer films: The saturation concentration of hexacyanoferrrate anions in (PAH-PGA)*_n_* films is of the order of 1 mol L^−1^ [68]. The same kind of polyelectrolyte multilayer films made from poly(L-glutamic acid) and poly(L-lysine) were loaded with potassium hexacyanoferrate and zinc meso tetrakis(sulfonatophenyl) porphyrin and the obtained composite film was able to reduce C_60_ in the organic solvent in contact with the immobilized dye and redox probe [75].

**Figure 7:**
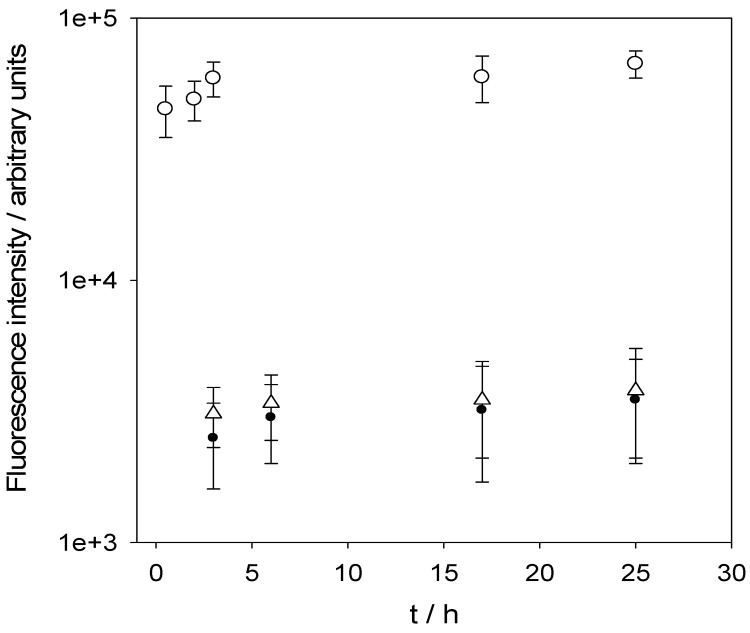
Release kinetics of CdTe nanoparticles (capped with thioglycolic acid) from (PDDA-PAA)_100_ films in the presence of a solution at pH 9 (○), at pH 7 (■) and at pH 9 after the deposition of a (PDDA-PSS)_10_ capping layer on top of the (PDDA-PAA)_100_ films filled with nanoparticles (25B3;). The error bars correspond to ± one standard deviation over six independent experiments. The release kinetics was measured by means of fluorescence spectroscopy of the solution in contact with the film. Reproduced with permission from [76]. Copyright 2008 the American Chemical Society.

The same concept of postloading of the as built films holds true for nanoparticles and proteins. In the case of CdTe quantum dots, a strong preferential incorporation in (PDDA-PAA)_100_ PEM films made from poly(diallyldimethylammonium chloride) (PDDA) and poly(acrylic acid) (PAA) was found for particles caped with the negatively charge thioglycolate with respect to the same particles coated with amino groups. In addition, the loading process was accompanied by an important film swelling. The reversible-irreversible character of the loading-release process could be controlled by the pH of the solution put in contact with the film (Figure 7) or by capping the exponentially growing and CdTe loaded film with an impermeable (PDDA-PSS)_10_ film [76] .

In the case of BSA labeled with FITC diffusing in (PLL-HA)*_n_* films, the protein concentration in the film can be very high, 75 mg mL^−1^ when the bulk concentration was varied between 0.05 and 1.2 mg mL^−1^. In addition, the protein’s diffusion coefficient (measured by means of fluorescence recovery after photobleaching) decreases progressively when the concentration of embedded protein is increased [77]. In addition, the fraction of highly mobile proteins decreases with an increase in the concentration of proteins incorporated in the film.

The same concept of spontaneous dye diffusion in polyelectrolyte multilayer films was used to show the possibility to remove toxic dyes present in waste water from the textile industry (methylene blue and coomassie brilliant blue). For this aim the substrates were coated with PEM films made from poly(ethylene imine) or chitosan as the polycations and from poly(acrylic acid) (PAA) as the polyanion [78].

These findings would tend to make the assumption that the thicker the polyelectrolyte based films, the higher the expected loading capacity for the dyes to be incorporated. This assumption relies on an homogeneous dye concentration across the thickness of the film. This is however not always the case as shown for the incorporation of CuPc-SO_4_ in multilayer films based on poly(sodium-L-glutamate) and poly(L-lysine hydrobromide) (Figure 6B) [73]. In addition, it has been found that the homogeneity of the multilayer film filling in the direction perpendicular to the substrate can depend on the solution concentration of the nanoparticles used to fill the film. Namely, in the case of polyoxoanions, the smaller the concentration in solution, the higher the average concentration of those nanoparticles in the film and the more homogeneous was their distribution across the film [79].

**Figure 8:**
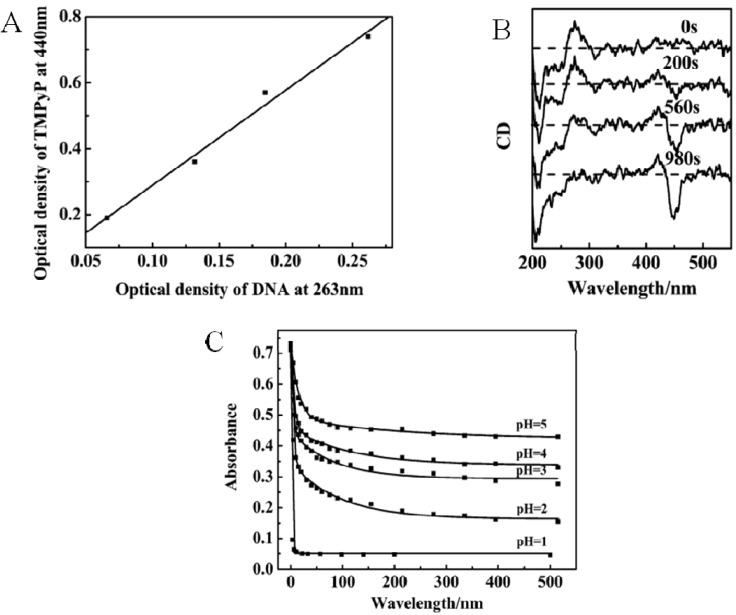
(**A**) Evolution of the absorbance due to the incorporated dye, TMPyP (at λ = 440 nm) as a function of DNA already present in the film (quantified by the absorbance at λ = 263 nm). The experiments were done by putting (PAN-DNA)*_n_* films in contact with 1 mM TMPyP at pH 8.4 during 10 min for films made from *n* = 7, 15, 23, 31 layer pairs. (**B**) Circular dichroism spectra of (PAH-DNA)*_n_*_=31_ films put in contact with a 1 mM TMPyP solution at pH = 8.4 for different time durations as indicated. (**C**) Release kinetics of TMPyP from (PAH-DNA)*_n_*_=31_ films. The films were loaded from 1 mM dye solutions at pH = 8.4 and subsequently put in contact with water at the indicated pH value and the release kinetics was followed by means of UV-Vis spectroscopy. Reproduced with permission from [72]. Copyright 2004 Elsevier.

Contrarily to the previous finding, (PAH-DNA)*_n_* films can be filled homogeneously through their whole thickness with the cationic dye 5,10,15,20 tetrakis (4-*N*-methylpyridyl) porphyrin-tetra-para(toluene sulfonate) (TMPyP). This was nicely shown by plotting the change in absorbance at 440 nm (due to TMPyP) *vs.* the change in absorbance at 261 nm (due to DNA) (Figure 8A) [72]. In this investigation, it was shown that part of the dye undergoes an induced circular dichroism, a negative Cotton effect at 453 nm (Figure 8B). This indicated that part of TMPyP intercalates in the DNA double helix. Further evidence for that came from the release kinetics: It could be fitted with the sum of two exponential decay functions. The slow release process corresponds to the TMPyP molecules intercalated in the DNA double helix whereas the fast process corresponds to the cationic dye bound to DNA through electrostatic interactions. The release rates as well the released amounts increased upon a decrease of the pH (Figure 7C) in contact with the film in relation with an increased protonation of the PAH polycation. The findings obtained for TMPyP diffusing and binding in (PAH-DNA)*_n_* films have been generalized to other dyes among which the DNA intercalantant ethidium bromide and acridine orange as well as mixtures of these dyes [80].

The optimization of the conditions allowed for an increase in the loading capacity of polyelectrolyte multilayer sponges for dyes has hence to be investigated in a more systematic manner with respect to the film thickness, dye concentration in solution, pH and ionic strength of the solution.

**Figure 9:**
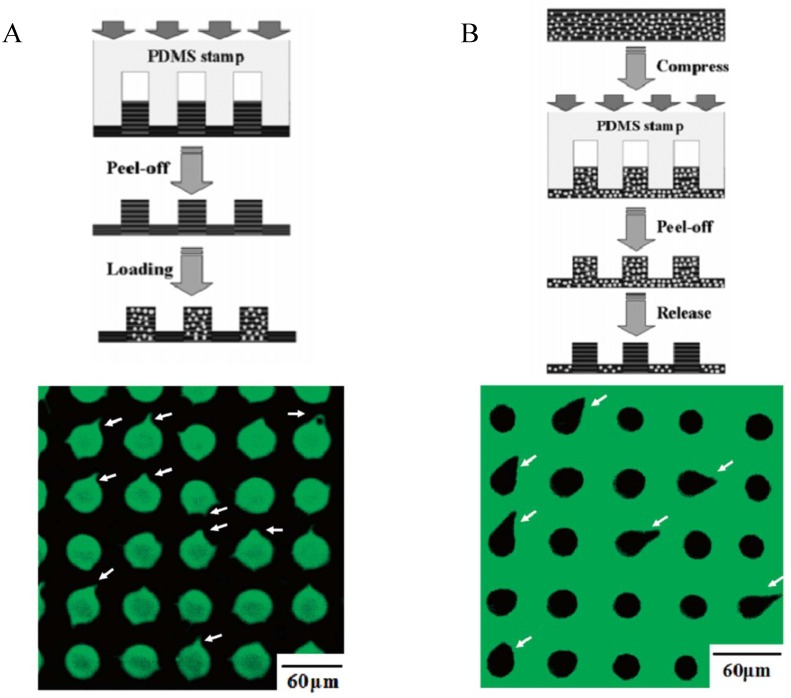
(**A**) Influence of local compression (applied using a periodic stamp made from PDMS) on the loading of a PEI-(PSS-PDDA)_7_ multilayer film with fluorescein. Only the uncompressed part of the films allow for important dye incorporation as shown in the fluorescence micrographs. (**B**) Loading of the PEI-(PSS-PDDA)*_n_* films preceded the application of local compression by the PDMS stamp and contact with an aqueous solution containing 2 mol L^−1^ NaCl. The dye remained localized in the compressed part of the films and leached out from the uncompressed parts. Reproduced with permission from [‎81]. Copyright 2005 the American Chemical Society.

The loading as well as the release of fluorescein and rhodamine 6G in PEI-(PSS-PDDA)_7_ films has been investigated in the case of uncompressed films as well as in the case of films that underwent compressive load (200 g cm^−2^ during 30 min) with periodic poly(dimethylsiloxane) (PDMS) stamps. It was found that the dyes diffused only in the uncompressed parts of the films (Figure 9A). This confirms the idea that permeation of dyes in polyelectrolyte multilayer films is possible only if there remains accessible pores to host the dyes, as suggested by Burke and Barrett [71]. Complementary, when the films are filled with the dyes before the application of the compressive load, the dye release kinetics is much faster and more important in the uncompressed parts of the films with respect to the compressed regions (Figure 9B) [81].

### 5.2. Exponentially Growing Polyelectrolyte Multilayers as Active Templates for the Synthesis of Inorganic Dyes

(PLL-HA)_10_ films display a positive Donnan potential and allow hence for the diffusion of the negatively charged and hydrosoluble Ti(IV) (bisammoniumlactato) dihydroxyde in the bulk of such films. The presence of free amino groups allowed for the de-coordination of lactic acid from Ti^4+^ and hence for the polycondensation of the titanium cations and for the formation of poorly crystalline titanium dioxyde [82].

## 6. Conclusions

Films obtained by the layer-by-layer deposition of charged species allow to concentrate dyes in an important manner with respect to their concentration in solution and to obtain the dye in a quasi solid state. There are three main ways to load the dyes in such films:

(i) By using charged dyes in the layering process. This layer-by-layer deposition of charged dyes and oppositely charged polyelectrolytes leads most often to dye aggregates in the films but also to an ordered assembly of well aligned dyes offering the possibility to use such films in optoelectronic applications.
(ii) By depositing polyelectrolytes carrying covalently bound dyes. In this case, the obtained films are highly stable because the dyes cannot diffuse spontaneously and allow for fascinating energy transfer processes as well as to investigate physicochemical properties of the multilayer films.
(iii) By allowing the dyes to diffuse in the polyelectrolyte multilayers films in a post deposition manner. Most often, the films display a high level of selectivity for the dyes according to the Donnan potential of the film and the charge of the dye. This selectivity can be easily switched for a given charge of the dye by playing on the solution pH. In turn after loading the dye in the film, its release kinetics in the solution can be controlled by the solution pH in the sense that it could allow for a partial uncharging of the polyelectrolyte having a high affinity, through electrostatic interactions, with the charged dye.

The loading of polyelectrolyte multilayer films with dyes according to this post-deposition process could offer nice opportunities for the removal of toxic dyes in the textile industry, provided the coatings remain stable upon dye loading. Indeed dye loading is most often accompanied by an important swelling of the film. In turn, this swelling process can lead to a loss of the film’s cohesion and its final decomposition [83]. Hence, to use polyelectrolyte multilayer films for dye depolution, more fundamental investigations are required to ensure the homogeneity of the film loading in the direction perpendicular to the substrate and its cohesion upon dye accumulation.
